# Using the Boston Syncope Observation Management Pathway to Reduce Hospital Admission and Adverse Outcomes

**DOI:** 10.5811/westjem.2018.11.39657

**Published:** 2019-02-04

**Authors:** Oren J. Mechanic, Celine Y. Pascheles, Gregory J. Lopez, Alina M. Winans, Nathan I. Shapiro, Carrie Tibbles, Richard E. Wolfe, Shamai A. Grossman

**Affiliations:** * Harvard Medical School, Beth Israel Deaconess Medical Center, Department of Emergency Medicine, Boston, Massachusetts; † Sidney Kimmel Medical College at Thomas Jefferson University, Philadelphia, Pennsylvania

## Abstract

**Introduction:**

In an age of increasing scrutiny of each hospital admission, emergency department (ED) observation has been identified as a low-cost alternative. Prior studies have shown admission rates for syncope in the United States to be as high as 70%. However, the safety and utility of substituting ED observation unit (EDOU) syncope management has not been well studied. The objective of this study was to evaluate the safety of EDOU for the management of patients presenting to the ED with syncope and its efficacy in reducing hospital admissions.

**Methods:**

This was a prospective before-and-after cohort study of consecutive patients presenting with syncope who were seen in an urban ED and were either admitted to the hospital, discharged, or placed in the EDOU. We first performed an observation study of syncope management and then implemented an ED observation-based management pathway. We identified critical interventions and 30-day outcomes. We compared proportions of admissions and adverse events rates with a chi-squared or Fisher’s exact test.

**Results:**

In the “before” phase, 570 patients were enrolled, with 334 (59%) admitted and 27 (5%) placed in the EDOU; 3% of patients discharged from the ED had critical interventions within 30 days and 10% returned. After the management pathway was introduced, 489 patients were enrolled; 34% (p<0.001) of pathway patients were admitted while 20% were placed in the EDOU; 3% (p=0.99) of discharged patients had critical interventions at 30 days and 3% returned (p=0.001).

**Conclusion:**

A focused syncope management pathway effectively reduces hospital admissions and adverse events following discharge and returns to the ED.

## INTRODUCTION

Prior studies have shown admission rates for syncope in the United States (U.S.) to be as high as 70%, triggering at least 2% of hospital admissions from the emergency department (ED) and 460,000 hospitalizations annually.^1^–^3^ Although emergency medicine has become more adept at distinguishing high-risk syncope from syncope of benign etiology and safety in ED discharge, there is a paucity of data addressing the care of patients once the ED recognizes a need for admission or further management.^4^–^17^ Recent data note that a typical hospital admission in the U.S. for syncope averages $5,300 with a total cost of syncope-related admissions of over $2 billion per year.^1^–^3^, ^16^–^21^ These costs have been directly related to the broad diagnostic testing performed to discover the etiologies of syncope.^20^ Not unexpectedly then, syncope was recently noted to be the leading diagnosis associated with payment denials by the Centers for Medicare and Medicaid Services.^22^

As short hospital-inpatient stays and hospital readmissions undergo increased scrutiny, ED observation units (EDOU) are increasingly being used as a low-cost alternative to inpatient hospitalization. While efforts to reduce unnecessary and expensive admission have generated clinical decision guidelines regarding the decision to admit, they have only begun to assess the value and yield of testing in syncope and have not fully assessed the utility of expedited care in an observation unit.^23^–^24^ The safety of substituting ED observation for in-house care in syncope has not been well studied. The objective of this study was to evaluate the utility and safety of an ED observation-based management pathway for the evaluation of patients presenting to the ED following a syncopal event.

## METHODS

### Study Design and Setting

This was a prospective cohort before-and-after study conducted in a large, urban teaching hospital with an annual ED census of 56,000 and an annual ED observation volume of approximately 6,000 visits. We performed an observational study of consecutive patients with syncope who were initially seen in the ED and were either admitted to the hospital, discharged or placed in an EDOU. We then implemented an ED-based, focused management pathway – the Boston Syncope Management Pathway (BSCMP) – to investigate the outcomes of these patients who presented to the ED with syncope (Figure). The BSCMP was derived by emergency physicians (EP) and cardiologists to create individualized workups for syncope based on presenting symptoms and comorbidities. The derivation used preexisting medical literature evaluating care of patients with syncope.^2^, ^9^, ^11^–^16^ Institutional review board approval was obtained prior to initiation of the study.

### Selection of Participants

Inclusion criteria were as follows: 1) age 18 years or older; and 2) ED patients presenting with syncope or near syncope and admitted by the ED team to either an inpatient ward or EDOU. We defined syncope as a sudden and transient (< five minutes) loss of consciousness producing a brief period of unresponsiveness and a loss of postural tone ultimately resulting in spontaneous recovery requiring no resuscitation measures.^9^, ^17^ Near syncope was defined as “feeling like they were going to pass out” but without actual loss of consciousness. Exclusion criteria were patients discharged home directly from the ED without an observation stay, patients with persistent altered mental status, alcohol or illicit drug-related loss of consciousness, seizure, coma, hypoglycemia, or transient loss of consciousness caused by head trauma.

**Population Health Research Capsule**

What do we already know about this issue?

*Although emergency department (ED) observation has been utilized for syncope, the safety and maximal utility of substituting ED observation for in-house care in syncope has not been well studied*.

What was the research question?

*This study aimed to evaluate the safety and effectiveness of an ED management Observation Pathway*.

What was the major finding of the study?

*A syncope management observation pathway reduced hospital admissions and adverse events, when compared to standard ED or inpatient care*.

How does this improve population health?

*With rising health care costs, hospital crowding, and increased ED boarding, a syncope management pathway is reliable, safe, and effective for ED patients*.

### Outcome Measures

The primary outcome was the utility of the BSCMP for the management of patients presenting to the ED with syncope. Secondary outcomes looked at the efficacy of the pathway in reducing hospital admissions and improving patient disposition. We defined significant events, as has been defined previously, to include critical interventions such as pacemaker or defibrillator placement, percutaneous coronary intervention, surgery, blood transfusion, cardiopulmonary resuscitation, endoscopy and carotid artery interventions, or adverse outcomes such as death, pulmonary embolus, myocardial infarction, cerebrovascular accident, dysrhythmia, cardiac arrest, intracranial hemorrhage or sepsis.^6^ We made secondary comparisons for patient demographics, comorbidities, and other features of their clinical presentation based on inpatient vs EDOU admission.

### Data Collection and Processing

An electronic ED dashboard that interfaced with a commercially available healthcare information system automatically tracked all ED patients, identifying and flagging those with complaints of syncope, near syncope or loss of consciousness for provider enrollment. In addition, the investigators routinely reviewed daily patient logs to ensure appropriate pathway enrollment and identify missed patients. The ED dashboard does not allow for a physician to place a patient disposition without enrolling (with a written explanation) or declining pathway placement. A chart review was then performed of these patients reviewing their ED and EDOU or hospital course. Finally, we recorded outcomes at 30 days following initial presentation to the ED mainly via medical record reviews and a few through phone calls.

### Primary Data Analysis

We entered data into a RedCap database. Categorical data was then analyzed using either chi-squared or Fisher’s exact test. We analyzed continuous data using Student’s t-test. Results are reported as percentages.

## RESULTS

Patient demographics and comorbidities pre- and post-pathway are described in Table 1. These show a slightly older population in the post-pathway group with significantly fewer signs of acute coronary syndrome or signs of conduction disease; however, they indicated more worrisome cardiac history, valvular heart disease, and abnormal vital signs. As described in Table 2, prior to implementation of the BSCMP, of the 570 patients enrolled, 344 (58.6%) were fully admitted and 27 (4.7%) were placed in the EDOU. A total of 209 (36.7%) patients were discharged immediately following ED evaluation. After the pathway was introduced, 489 patients were enrolled. Of the 489 patients enrolled, 164 (33.5%) were admitted and 96 (19.6%) were placed in the EDOU. More patients were discharged directly from the ED to home in the post-pathway vs pre-pathway studies (36.7% vs 46.8%; p<0.001). The observation unit post-BSCMP managed 96 (19.6%) patients presenting to the ED for syncope vs 27 (4.7%) prior to pathway implementation (p<0.001). Of the patients placed in the EDOU, 11 (11.4%) were admitted from the EDOU.

As described in Table 3, of the 209 discharged patients from the ED, prior to the management pathway 21 (10%) returned to the ED for syncope. In comparison to the post-pathway cohort, only six (2.6%) re-presented to the ED for syncope after discharge (p=0.001). Although return visits decreased among discharged patients post pathway, 30-day adverse events were similar for these groups. Pre-pathway, 30-day adverse events were 3% (6/209) vs 3% (7/229; p=0.99) post-pathway. Table 4 describes the pre- and post-pathway 30-day return diagnoses post-discharge for syncope.

## DISCUSSION

Our data suggest that a focused syncope management pathway may effectively reduce hospital admissions without increasing adverse events following discharge. EDOUs were designed to provide focused care in lieu of admission, with an expectation of discharge within 24 hours. The utility of ED observation has long been established for patients with diagnoses such as chest pain, asthma, congestive heart failure, and cellulitis, which in the past would often result in short hospital stays.^25^–^29^ Like chest pain, syncope is a common presentation that uncommonly signifies a dangerous, underlying condition and should be amenable to this approach.

The BSCMP was designed to direct care and refocus EPs not only in differentiating life threats from less-dangerous causes of syncope but to enable the EP to selectively manage those patients with potential risk factors for adverse event. To do so, the pathway directs physicians toward testing in fixed circumstances and to discharge patients who are low risk based on the BSCMP.^6^ Lastly, if neither the EDOU nor discharge is appropriate, the pathway recommends admission. This, in turn, likely reflects the broad spectrum of diseases that syncopal etiologies span, from potentially life-threatening to low-risk diagnoses.

A prior study comparing an ED observation syncope protocol and routine inpatient admission found that observation reduced admission rates and hospital length of stay with no differences in 30-day quality-of-life scores or patient satisfaction.^30^,^31^ This study also suggested a reduction in hospital costs, with no difference in safety.^30^ We believe the BSCMP takes this one step further, as our data suggest not only a reduction in admission rates but a significant decrease in the number of returns and readmissions to the hospital for syncope patients. Given the financial constraints involved in the current healthcare climate, this finding becomes increasingly significant.

While fewer than one-third of EDs currently have EDOUs, this number is growing and our ability to adequately care for these growing patient populations needs to be commensurate.

## LIMITATIONS

There are a number of limitations in this study, including possible selection bias in assigning patients to observation units vs inpatient admission. Additionally, the demographics were different: The pre-pathway population was younger and had clearly different risk factors than the post-pathway group. We also used a single institution for a test site, where the use of the BSCMP is well engrained as a practice guideline. This results in a lack of generalizability of the conclusions of this study. The sample size of this study was small, and there was lack of long-term follow-up >30 days in these patients. For each adverse outcome that was reported, discerning an outcome as causative may not always be uniform.

## CONCLUSION

A focused syncope management pathway may effectively reduce hospital admissions and, in turn, minimize adverse events following discharge and potentially decrease the total number of returns to the ED in the ensuing 30 days.

## Figures and Tables

**Figure:**
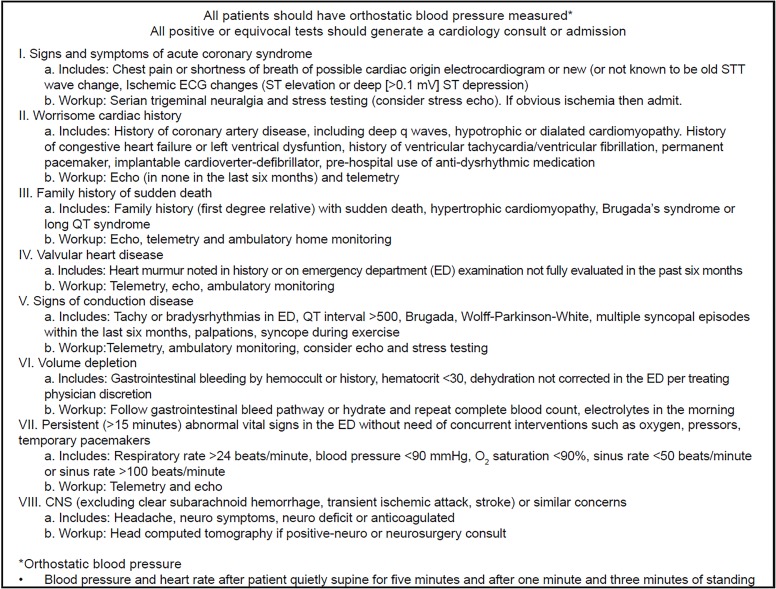
Boston Syncope Pathway to guide the management of patients with syncope in the emergency department. This is a validated pathway for the management of syncope in the ED.^6^

**Table 1:** Patient demographics and risk factors for adverse outcomes in syncope; pre and post-pathway.

	Pre-pathway	Post-pathway	P value
Number of patients	570	489	-
Age, mean (SD)	53.6 (24.2)	56.7 (22.8)	0.03
Male, % (n)			
Risk factors			
Signs of ACS (chest pain, ischemic, SOB, abnormal heart rhythm)	26.1% (149)	13.5% (66)	<0.001
Signs of conduction disease (recurrent syncope, palpitations, syncope with exercise, QT > 500 ms, heart block)	13.5% (77)	8.4% (41)	<0.01
Worrisome cardiac history (CAD, CHF, V-tach, pacemaker, ICD)	33% (188)	41% (201)	<0.01
Valvular heart disease (i.e. significant murmur)	4% (23)	7% (35)	0.03
Family history of sudden death	2% (11)	0.8% (4)	0.19
Persistent abnormal vital signs in ED (RR>24, O2<90, HR<50 or >100, SBP<90)	6.5% (37)	17% (83)	<0.001
Volume depletion (GIB, Hct < 30, profound dehydration)	6% (34)	8% (38)	0.24
Primary CNS event	1% (7)	2% (12)	0.17

*SD*, standard deviation; *ACS*, acute coronary syndrome; *SOB*, shortness of breath; *CAD*, coronary artery disease; *CHF*, congestive heart failure; *V-tach*, ventricular tachycardia; *ICD*, implantable cardioverter-defibrillator; *ED*, emergency department; *SBP*, systolic blood pressure; *HR*, heart rate; *GIB*, gastrointestinal bleed; *Hct*, hematocrit; *CNS*, central nervous system.

**Table 2:** Comparison of pre-pathway and post-pathway admission, emergency department observational (ED Obs), and discharged patients.

	Pre-pathway	Post-pathway	P value
Number of patients	570	489	-
Admitted	58.6%(334)	33.5% (164)	p<0.001
ED Obs	4.7% (27)	19.6% (96)	p<0.001
Discharged	36.7% (209)	46.8 (229)	p<0.001

**Table 3:** Return visits to the emergency department (ED) and 30-day adverse events (AE).

	Pre-pathway	Post-pathway	P value
Discharged	209	229	-
Return ED Visit	10% (21)	2.6% (6)	0.001
30-Day AE	3% (6)	3% (7)	p<0.99

**Table 4:** Description of return adverse events after discharge.

Pre-pathway (n=6/209) discharged	Post-pathway (n = 7/229) discharged
Myocardial infarction= 1	Anemia requiring transfusion= 1
PCI/surgery= 1	Vaginal bleed= 1
Ventricular dysrhythmia= 1	Ventricular tachycardia= 1
GI bleed=1	Death= 1
PE= 1	Surgery= 3
Sepsis= 1	

*PCI*, percutaneous coronary intervention; *GI*, gastrointestinal; *PE*, pulmonary embolism.
